# The Absence of Gut Microbiota Alters the Development of the Apicomplexan Parasite *Eimeria tenella*

**DOI:** 10.3389/fcimb.2020.632556

**Published:** 2021-02-04

**Authors:** Pauline Gaboriaud, Guillaume Sadrin, Edouard Guitton, Geneviève Fort, Alisson Niepceron, Nathalie Lallier, Christelle Rossignol, Thibaut Larcher, Alix Sausset, Rodrigo Guabiraba, Anne Silvestre, Sonia Lacroix-Lamandé, Catherine Schouler, Fabrice Laurent, Françoise I. Bussière

**Affiliations:** ^1^ INRAE, Université de Tours, UMR ISP, Nouzilly, France; ^2^ INRAE, UE PFIE, Nouzilly, France; ^3^ INRAE, Oniris, PAnTher, APEX, Nantes, France

**Keywords:** *Eimeria tenella*, microbiota, germ-free, chicken, parasite, parasite invasion and development

## Abstract

Coccidiosis is a widespread intestinal disease of poultry caused by a parasite of the genus *Eimeria. Eimeria tenella,* is one of the most virulent species that specifically colonizes the caeca, an organ which harbors a rich and complex microbiota. Our objective was to study the impact of the intestinal microbiota on parasite infection and development using an original model of germ-free broilers. We observed that germ-free chickens presented significantly much lower load of oocysts in caecal contents than conventional chickens. This decrease in parasite load was measurable in caecal tissue by RT-qPCR at early time points. Histological analysis revealed the presence of much less first (day 2pi) and second generation schizonts (day 3.5pi) in germ-free chickens than conventional chickens. Indeed, at day 3.5pi, second generation schizonts were respectively immature only in germ-free chickens suggesting a lengthening of the asexual phase of the parasite in the absence of microbiota. Accordingly to the consequence of this lengthening, a delay in specific gamete gene expressions, and a reduction of gamete detection by histological analysis in caeca of germ-free chickens were observed. These differences in parasite load might result from an initial reduction of the excystation efficiency of the parasite in the gut of germ-free chickens. However, as bile salts involved in the excystation step led to an even higher excystation efficiency in germ-free compared to conventional chickens, this result could not explain the difference in parasite load. Interestingly, when we shunted the excystation step *in vivo* by infecting chickens with sporozoites using the cloacal route of inoculation, parasite invasion was similar in germ-free and in conventional chickens but still resulted in significantly lower parasite load in germ-free chickens at day 7pi. Overall, these data highlighted that the absence of intestinal microbiota alters *E. tenella* replication. Strategies to modulate the microbiota and/or its metabolites could therefore be an alternative approach to limit the negative impact of coccidiosis in poultry.

## Introduction

*Eimeria* is an obligate intracellular parasite belonging to the phylum of Apicomplexa. In birds, various *Eimeria* species colonize the intestine with a localization that is specific to each species. *Eimeria tenella* (*E. tenella*) colonizes the caeca and is one of the most virulent species. It leads to haemorrhagic diarrhoea and high levels of morbidity and mortality in poultry flocks. Prevention and treatment of *Eimeria* infection are responsible for very high economic cost recently re-assessed to the level of about 13 billion dollars per year in the world (Blake et al., 2020). Current prophylaxis is based on anticoccidial drugs and vaccination, both with its advantages and limitations.

*E. tenella* invades and develops in intestinal epithelial cells. The *in vivo* parasite life cycle is divided in two parts: the endogenous asexual multiplication with three rounds of merogony and the sexual phase with the formation of macrogametes (female gametocytes) and microgametes (male gametocytes). Microgametes may fertilize a macrogamete resulting in the formation of a zygote (Ferguson et al., 2003). After maturation, it becomes an unsporulated oocyst released in the environment through the feces where it will develop into an infectious sporulated oocyst (exogenous phase or sporogony). Caeca are the richest part of the intestine in terms of bacteria abundance and diversity. Firmicutes, Bacteroidetes, and Proteobacteria are the main caecal bacterial phyla (Wei et al., 2013; Oakley et al., 2014; Qi et al., 2019). Importantly, *E. tenella* infection leads to alterations in the microbiota diversity and composition (Huang et al., 2018a). Taxa belonging to the order *Enterobacteriaceae* from the phylum Proteobacteria were increased in caecal contents from *E. tenella* infected chickens compared to those uninfected. Within the phylum Firmicutes, non-pathogenic bacteria *Lactobacillus* and *Faecalibacterium* were decreased while bacteria such as *Clostridium perfringens* are increased during the infection with *E. tenella* and are related to an increased occurrence of necrotic enteritis of chickens (Al-Sheikhly and Al-Saieg, 1980; Macdonald et al., 2017; Huang et al., 2018a; Huang et al., 2018b). The microbiota promotes the gut immune system maturation which in turn protect the host from enteric bacterial infections such as *Salmonella* infection (Crhanova et al., 2011). Studies have shown that the modulation of the chicken intestinal microbiota can limit *Salmonella* colonization (Stecher et al., 2010; Yang et al., 2014). In the case of an apicomplexan parasite such as *Plasmodium falciparum*, when *Enterobacter* is present in the gut, it confers a resistance to the parasite infection in *Anopheles* (Cirimotich et al., 2011a; Cirimotich et al., 2011b). However, other pathogens such as *Histomonas meleagridis* depend on bacteria for their growth *in vitro* (Ganas et al., 2012). Concerning *E. tenella*, most of the studies have focused on the effect of the microbiota on the clinical signs associated with infection (Visco and Burns, 1972a; Visco and Burns, 1972b; Bradley and Radhakrishnan, 1973; Lafont et al., 1975).

In the present study, we investigated the influence of the absence of microbiota on *E. tenella* infection. For this purpose, we used a recently developed model of germ-free fast-growing broilers (Guitton et al., 2020). Our results highlight a critical role for the microbiota in sustaining the optimal development of the parasite in the chicken intestinal tract. Strategies to modulate the composition of the microbiota and/or derived metabolites might therefore represent a promising strategy to improve the prophylaxis of coccidiosis.

## Materials and Methods

### Ethical Statement

Animal experimental protocols were performed in accordance with the French legislation (Décret: 2001‐464 29/05/01) and the EEC regulation (86/609/CEE) about laboratory animals. All chicken experiments were approved by the local ethics committee for animal experimentation of Centre Val de Loire (CEEA VdL n°19): 2018‐04‐26 (APAFIS N°13904).

### Parasite

The *E. tenella* INRAE PAPt36 strain (*Et*-INRAE) was used for all experiments except for studying the effect of the microbiota on parasite invasion. *E. tenella* strain was isolated from a poultry farming in France in 1974. Initially referenced as *E. tenella* strain PAPt38 is now renamed as *E. tenella*-INRAE strain. The strain was maintained by regular passages on chicken in the PFIE facility since then (Laurent et al., 2001). For parasite invasion studies, *Et*-INRAE was transfected with *nano luciferase* and *mcherry* genes under the *E. tenella actin* promoter [*Et*-INV; (Yan et al., 2009; Swale et al., 2019)]. The *mcherry* gene allows sorting by flow cytometry of sporulated oocysts after inoculation of transfected sporozoites to chickens and for parasite amplification. The nano luciferase activity (NanoLuc^®^ Luciferase, Promega) facilitates the detection of low load of parasites and accurate parasite quantification in tissues. Purification of sporozoites was performed as described by (Shirley, 1995). Briefly, 0.5 mm sterilized glass beads (Carl Roth, Karlsruhe, Germany) were added to sporulated oocysts. The oocyst wall was broken by vortexing for 17 s. Released sporocysts were washed with PBS and incubated in standard excystation medium (trypsin 0.25% and biliary salts 0.5% in PBS; pH 7.4) at 41°C for 1 h. Sporozoites were then washed in PBS and ready for cloacal inoculation.

### Animals

Conventional and germ-free Ross PM3 broilers were hatched in the Infectiology of Farm, Model and Wildlife Animals Facility (PFIE, Centre INRAE Val De Loire: https://doi.org/10.15454/1.5572352821559333E12; member of the National Infrastructure EMERG’IN) as described by (Guitton et al., 2020). Briefly, Ross PM3 eggs from two French farms were collected, decontaminated with a 1.5% peracetic acid solution (1.5% Divosan Plus VT53, Johnson Diversey, France). Eggs were then incubated, decontaminated a second time as described above and placed in a hatching incubator for production of conventional chicks or an isolator for the production of germ-free animals. Bacteriological controls were performed as described by (Guitton et al., 2020); animals were confirmed to be bacteria-free while conventional chickens developed a microbiota.

### Infection

Two-week-old chickens were orally infected with different doses of sporulated oocysts of the *Et*-INRAE strain. At day 6 to 9 post-infection, chickens were euthanized by electronarcosis and caeca were collected. On caecal contents, oocysts were counted on MacMaster counting chambers. At day 2, 3.5, 5.5, and 7 post-infection, caecal tissues were washed and fixed in 4% formaldehyde (Laurypath, Chaponost, France) for histological analysis or directly frozen in liquid nitrogen and stored at -80°C for gene expression analysis.

To study parasite invasion, the *Et*-INV strain was used. In order to shunt the natural excystation step *in vivo*, chickens were artificially infected by the cloacal route with 10^6^ sporozoites. After 16 h of infection, chickens were euthanized; caeca were removed and washed for measurement of the nano-luciferase activity (Promega). To investigate the parasite development in these conditions, chickens were cloacally infected with a lower dose of *Et*-INRAE sporozoites (8 x 10^4^). At day 7 post-infection, chickens were euthanized and caeca were removed. Oocyst numbers in caecal contents were determined and caecal tissues were fixed or frozen as described above.

### Sporozoite Excystation Test

For *ex vivo* sporozoite excystation test, the bile from the gall bladder and intestinal contents from different segments of the intestine of non-infected germ-free and conventional non-infected chickens were collected. Only intestinal contents were centrifuged at 7,000 g for 15 min. Intestinal supernatants (dilution 1/10 in PBS) and bile (dilution 1/100 in PBS) were incubated at 41°C for 30 min to 1h30 with sporocysts obtained as described by (Shirley, 1995). As a control, the standard excystation medium described above was used. The number of sporocysts and excysted sporozoites were counted and the excystation efficiency was calculated as follows: (number of excysted sporozoites/2)/[(number of excysted sporozoites/2) + number of sporocysts)] x 100.

### Histological Analysis

Caecal tissues were collected, fixed in 4% formaldehyde (Laurypath) and embedded in paraffin wax (Leica). Tissue sections were stained with kit Masson Trichrome, light green variation (RAL diagnostics) or Hemalun Eosin Saffron.

### RT-qPCR

Total RNA was isolated from caeca using TRIzol extraction (Life Technologies, Carlsbad, CA, USA). cDNA was synthetized using M-MLV Reverse Transcriptase (Promega, Madison, WI, USA), with random hexamer primers and oligo(dT)15 primer (Promega). cDNAs were then amplified by qPCR (CFX96, Bio-Rad, Hercules, CA, USA) using iQ™ SYBR^®^ Green Supermix (Bio-Rad). Parasite load into caecal tissues was determined using specific primers to the *E. tenella* housekeeping genes *Et18S*, *Etprofilin* and *Gallus gallus* housekeeping genes *g10* and *gapdh* (**Table 1**). *Et18S* is commonly used as a housekeeping gene during the parasite cycle (Walker et al., 2015); we added *Etprofilin* for which the expression was not modified in our experiment (data not shown). Microgamete and macrogamete gene expressions were determined using *Etfoa1*, (microgamete-specific gene; ETH_00025255) and *Etgam56*, (macrogamete-specific gene, ETH_00007320) both obtained from ToxoDB release 34 (**Table 1**; Eurogentec, Seraing, Belgium) and the previously cited *E. tenella* housekeeping genes. The protocol used for qPCR was: 95°C for 5 min and 40 cycles at 95°C for 10 s and 60°C for 15 s followed by 60°C for 5 s. Melting curves were performed at 60°C for 5 s followed by gradual heating (0.5°C/s) to 95°C. qPCR were performed in duplicate for each experiments. For parasite load, expression of *Et18S* and *Etprofilin* were normalized to Ct values obtained for *Gallus gallus g10 and gapdh* using the formula: 2^-(Ct mean of^ *^Et housekeeping genes^* ^– Ct mean of^ *^gallus gallus housekeeping^* ^genes)^. For parasite microgamete and macrogamete gene expression, *Etgam56* and *Etfoa1* were normalized to Ct values obtained for *Et18S and Etprofilin*RNA using the formula: 2^-(Ct^ *^Et gamete specific gene^* ^– Ct^ *^mean of Et housekeeping genes^* ^)^. Gene expression values are expressed as medians.

**Table 1:** Sequences of primers used for RT-qPCR.

Primer name	Accession number	Forward sequence (5' to 3')	Reverse sequence (5' to 3')
*Et18S*	18S rRNA gene in Supercontig190	CTGATGCATGCAACGAGTTT	GACCAGCCCCACAAAGTAAG
*Etprofilin*	ETH_00010645	GGAAGACGGAACCTCCATTT	CCAGAATCGCCACATCATAG
*Etfoa1*	ETH_00025255	TCTCGCATTCCTCACAGATG	ATTTCGCCTTGTGGATGAAC
*Etgam56*	ETH_00007320	AGTGGCTGGAGAACTTCGTG	ATGCGGTTCGTGATCATGTC
*Gallus gallus g10*	416492	TCAAGGAAGGGTACGCTACA	AACAGCCTCTGCATCCACAGT
*Gallus gallus gapdh*	374193	GTCCTCTCTGGCAAAGTCCAAG	CCACAACATACTCAGCACCTGC

### Nano Luciferase Activity

Caecal tissues were weighted, placed in lysis buffer (Tris 50 mM, EDTA 2 mM, Glycerol 10%, Triton X-100 1%) for 30 min at 4°C and then homogenized. After centrifugation (750 g, 5 min), 25 µl of supernatant were transferred to a 96-well plate and 25 µl of buffer with substrate (1/50) was added (Nano-Glo**^®^** Luciferase Assay System, Promega). Nano luciferase activity was measured using GloMax**^®^**-Multi Detection plate reader (Promega).

### Statistical Analysis

Data were analyzed using GraphPad Prism^®^ 6 (GraphPad Software Inc., La Jolla, CA, USA). For data with only two groups of animals, a non-parametric Mann-Whitney test was performed. When more than two groups of chickens were compared, a non-parametric ANOVA followed by Dunn’s multiple comparisons test was performed. When repeated measurements were performed, a non-parametric two-way ANOVA with multiple comparisons test was performed.

## Results

### Effect of the Absence of Microbiota on Oocyst Load in Caecal Contents and on Parasite Developmental Stages in Caecal Tissue

**Figure 1:**
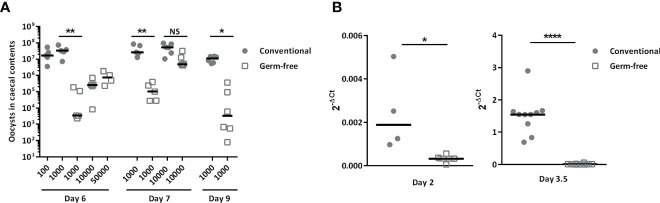
The absence of microbiota reduced parasite load in caecal contents and in caecal tissues during infection. Conventional and germ-free chickens were orally infected with different doses of *E. tenella*. **(A)** Oocyst load in caecal contents. Oocyst load was evaluated at day 6, 7, and 9 post-infection. Medians are represented (One-Way ANOVA, Dunn’s multiple comparisons test; n ≥ 4; *P < 0.05; **P < 0.01 between germ-free and conventional chickens). **(B)** The parasite load in caecal tissues is lower as soon as day 2 post-infection in germ-free chickens compared to conventional chickens. To be able to detect the parasite, conventional and germ-free chickens were orally infected with high doses of *E. tenella* (500,000 sporulated oocysts) for 2 days and 3.5 days of infection. The parasite load in caecal tissues was evaluated by RT-qPCR using the mean transcript expression of *Et18S* and *Etprofilin* relative to host housekeeping genes (*Gallus gallus gapdh* and *g10*) using the 2^-ΔCt^ formula: Ct *Et* housekeeping genes – Ct host housekeeping genes. Medians are represented (Mann-Whitney; n ≥ 4; *P < 0.05; ****P < 0.0001 between germ-free and conventional chickens).

To study the absence of microbiota on *E. tenella* infection, germ-free and conventional ROSS PM3 fast-growing broilers were infected with different doses of oocysts. The animals were euthanized and oocysts were counted in caecal contents at day 6, 7, and 9 days post-infection. We counted oocysts in caecal contents as it was technically not feasible to collect faeces daily for oocysts counts in our isolators and to maintain germ-free conditions concomitantly. Oocyst load were severely decreased in the caeca of germ-free chickens inoculated *per os* with a dose of 1,000 oocysts compared to conventional chickens at these different time points (day 6: 1,192-fold decrease; day 7: 269-fold decrease; day 9: 129-fold decrease). Remarkably, decreasing the dose of inoculum to 100 oocysts for conventional animals was not enough to decrease their parasite load in caecal contents to the level of germ-free animals 6 days post-infection when they were inoculated *per os* 1,000 or 10,000 or even 50,000 sporulated oocysts (**Figure 1A**). However, inoculation of 10,000 sporulated oocysts led to a similar parasite load in caecal contents at day 7 post-infection for both germ-free and conventional chickens (**Figure 1A**).

**Figure 2:**
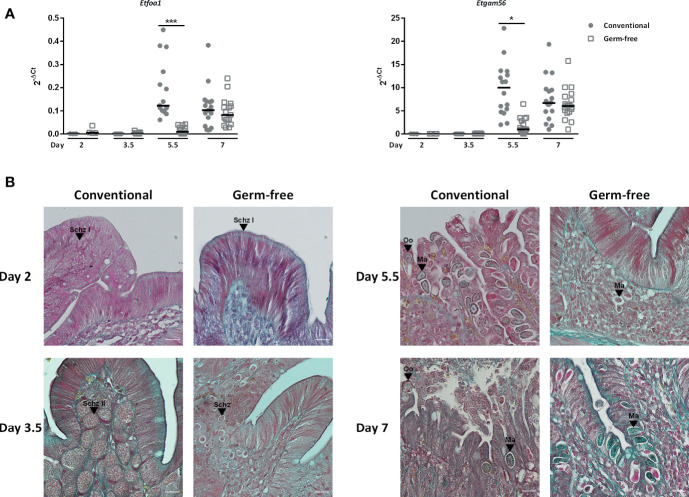
The presence of immature second generation schizonts in germ-free chickens at day 3.5 post-infection suggests a delay in the development of the parasite. **(A)** Gametocyte gene expressions are delayed in germ-free chickens. Sporulated oocysts were orally inoculated to germ-free and conventional chickens (500,000 for 2 days and 10,000 for 3.5, 5.5, and 7 days of infection). Gametocyte gene expressions were detected by RT-qPCR on caecal tissues. The expression of microgamete and macrogamete specific genes (*Etfoa1* and *Etgam56)* were determined by RT-qPCR and expressed relative to *E. tenella* housekeeping genes (*Et18S* and *Etprofilin*) at day 2, 3.5, 5.5, and 7 post-infection using the 2^-ΔCt^ formula: Ct *Et* gametocyte specific gene – Ct *Et* housekeeping genes. Medians are represented (One-Way ANOVA, Dunn’s multiple comparisons test; n ≥ 3; *P< 0.05; ***P <0.001 between germ-free and conventional chickens). **(B)** Representative histological images showing a delay in parasite development in germ-free chickens. Histological analysis was performed on tissue sections stained with Masson-Goldner Trichrome. Scale = 20 µm; Schz, schizont; Ma, macrogamete; Oo, oocyst.

Interestingly, lower parasite load in caecal tissues of germ-free chickens was already observed as soon as day 2 post-infection (**Figure 1B**). Indeed, histological analysis revealed less first-generation schizonts at day 2 post-infection and less second-generation schizonts at day 3.5 post-infection in germ-free compared to conventional chickens. At this later time point, second-generation schizonts were clearly smaller, immature and second generation merozoites were not formed yet in germ-free chickens compared to conventional chickens in which the second-generation schizonts were mature with clearly developed merozoites (**Figure 2B**). This observation suggests a delay in the beginning of the parasite replication phase in the absence of microbiota (**Figure 2B**). We next studied the expression of gamete specific genes *Etfoa1* (for microgamete) and *Etgam56* (for macrogamete) (Walker et al., 2015). For this purpose, gamete gene expressions were normalized to the parasite housekeeping genes (*Etprofilin* and *Et18S*). We observed reduced *Etfoa1* and *Etgam56* gene expressions in germ-free compared to conventional chickens at day 5.5 post-infection (**Figure 2A**). This result was corroborated by histological analysis showing a higher number of parasites in the stage of gametes in conventional compared to germ-free chickens at this time point (**Figure 2B**). At day 7 post-infection, the expression of both *Etfoa1* and *Etgam56* were similar for both groups (**Figure 2A**). Histological analysis revealed that the number of gametes decreased in conventional chickens at day 7 post-infection (**Figure 2B**) and increased in germ-free chickens. These data suggest a delay in *E. tenella* development as a result of a longer asexual phase in germ-free compared to conventional chickens.

### Bile From Germ-Free Chickens Increases Sporozoite Excystation

**Figure 3:**
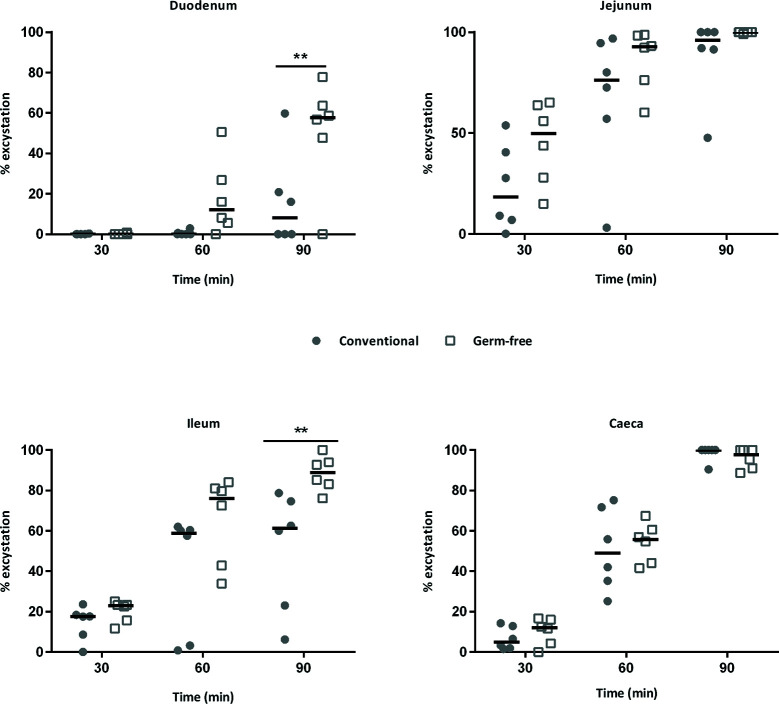
The sporozoite excystation efficiency is increased with bile and duodenum or ileum contents from germ-free chickens. Sporozoites were excysted using contents of different segments of the intestine and bile from the same animals (germ-free and conventional chickens). The excystation efficiency of sporozoites is higher when incubating sporocysts with duodenal, ileal contents and bile from germ-free chickens. Medians are represented (Two-Way ANOVA, Sidak multiple comparisons test; n = 6; **P < 0.01 between germ-free and conventional chickens).

**Figure 4:**
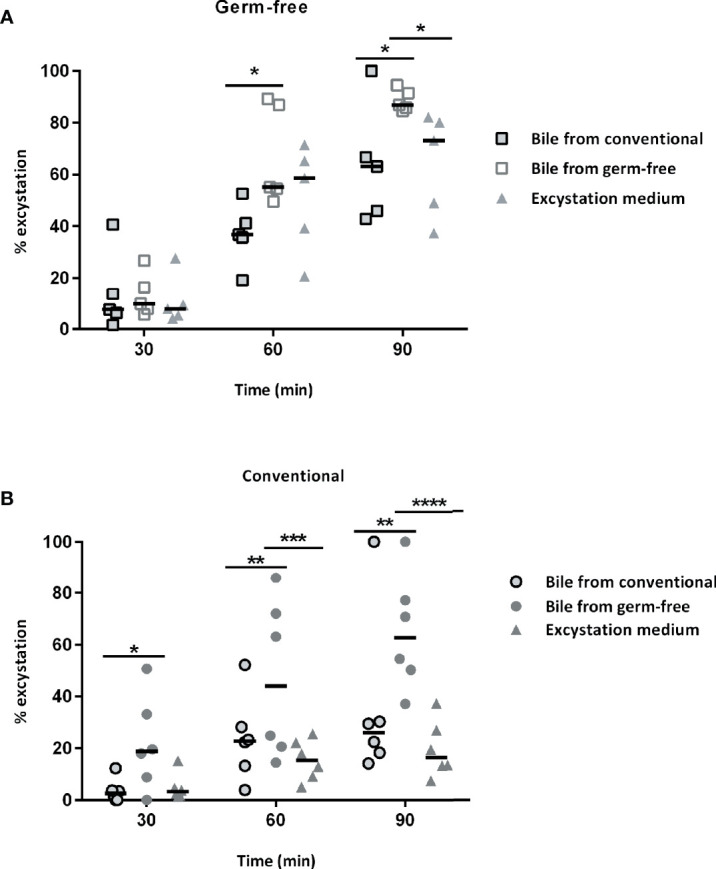
The bile from germ-free chickens is responsible for the increase in the sporozoite excystation efficiency. **(A)** Bile from germ-free chicken leads to a higher excystation efficiency of sporozoites when incubating sporocysts with duodenal contents from germ-free chickens. Sporozoites were excysted when incubating sporocysts with duodenal contents from germ-free chickens and bile from conventional, germ-free chickens or excystation medium. Medians are represented (Two-Way ANOVA, Tukey multiple comparisons test; n = 5; *P < 0.05). **(B)** Bile from germ-free chicken leads to a higher excystation efficiency of sporozoites when incubating sporocysts with duodenal contents from conventional chickens. Sporozoites were excysted when incubating sporocysts with duodenal contents from conventional chickens and bile from conventional, germ-free chickens or the standard excystation medium. Medians are represented (Two-Way ANOVA, Tukey multiple comparisons test; n = 6; *P < 0.05; **P < 0.01; ***P < 0.001; ****P < 0.0001).

When sporulated oocysts are ingested by chickens, mechanic and enzymatic activities such as trypsin and biliary salts are necessary for sporozoite excystation. Sporozoites are then able to invade intestinal epithelial cells in which the parasite develops. Since lower parasite development in germ-free chickens may have resulted from a decrease in the excystation efficiency, we investigated the ability of sporozoites to excyst in the presence of intestinal contents and bile extracts from conventional and germ-free chickens. We then tested the activities of intestinal contents from specific segments of the gut and bile obtained from the gall bladder from germ-free and conventional chickens. We observed a higher excystation efficiency with duodenal and ileal supernatants combined with bile from germ-free chickens (**Figure 3**). To clarify the respective contribution of the intestinal content and the bile in this higher excystation efficiency, we used duodenal contents from germ-free or conventional chickens supplemented with bile from conventional, germ-free, or the standard excystation medium as a control. In these conditions, we observed a higher excystation efficiency when using duodenal contents from germ-free or conventional chickens combined with bile from germ-free chickens (**Figures 4A, B** **)**. This result demonstrates that the higher excystation efficiency was likely to be related to the bile composition of germ-free chickens. However, this data cannot explain the lower parasite load observed caecal contents from germ-free compared to conventional chickens as a higher efficiency of excystation in germ-free chickens would be expected to lead to a higher infection rate.

### The Absence of Gut Microbiota Alters the Development of the Parasite but Not Its Capacity to Invade the Caecal Mucosa

**Figure 5:**
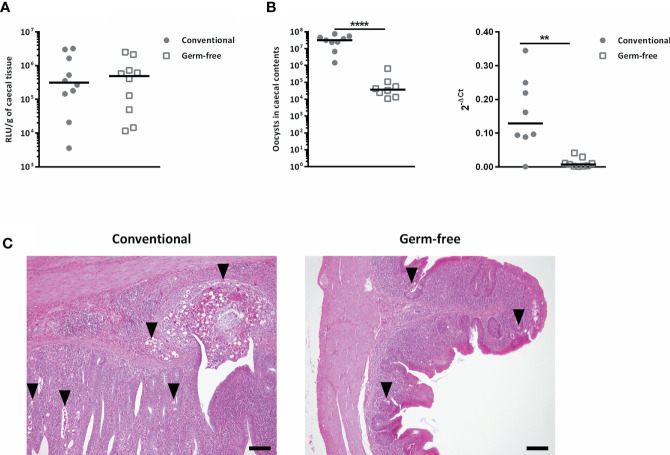
The absence of gut microbiota has no effect on *E. tenella* sporozoite invasion capacity but alters its development. **(A)** The absence of microbiota had no effect on *E*. *tenella* sporozoite invasion capacity. Conventional and germ-free chickens were cloacally inoculated with *E. tenella* sporozoites expressing a *nano luciferase* reporter gene (10^6^ sporozoites per animal). Caecal tissues were washed in PBS and luciferase activity was detected 16 h post-infection. Medians are represented (Mann-Whitney; n = 10; non-significant). No significant differences between germ-free and conventional chickens were observed. **(B)** The absence of microbiota alters the development of *E. tenella*. Conventional and germ-free chickens were cloacally inoculated with *Et*-INRAE sporozoites (8 x 10^4^). *On the left panel*: Oocysts were counted in caecal contents at day 7 post-infection. *On the right panel*: the parasite load in caecal tissues at day 7 post-infection was determined by RT-qPCR. The expression of housekeeping parasite specific genes, *Et18S* and *Etprofilin* were expressed relative to *Gallus gallus* housekeeping genes *g10* and *gapdh* using the 2^-ΔCt^ formula: Ct *Et* housekeeping genes – Ct host housekeeping genes. Medians are represented (Mann-Whitney; n ≥ 8; **P < 0.01; ****P < 0.0001 between germ-free and conventional chickens). **(C)** Representative histological images at day 7 post-infection showing lower parasite load in caecal tissues in germ-free chickens compared to conventional chickens. Histological analysis was performed on tissue sections stained with Hemalun Eosin Saffron; scale = 100 µm.

In order to study the effect of the absence of microbiota on sporozoite invasion *in vivo*, without the influence of the bile on the excystation efficiency, we shunted this process by cloacally infecting chickens with sporozoites excysted *in vitro*. Using *E. tenella* sporozoites expressing the *nano luciferase* reporter gene, we were able to determine parasite invasion level and showed that there was no difference in parasite invasion between conventional and germ-free chickens (**Figure 5A**). However, in the same experimental conditions when the animals were kept until day 7 post-infection, a decrease in oocyst load in caecal contents (280-fold) and in tissue parasite load revealed by RT-qPCR and histological analysis were observed in germ-free compared to conventional chickens (**Figures 5B, C** **)**. These results suggest a major role of the microbiota on the development of the parasite after the invasion process but not on the parasite invasion itself.

## Discussion

To study the effect of the microbiota on *E. tenella* infection, we used an original model of germ-free broilers that we recently developed in our facilities (Guitton et al., 2020). A previous study reported that a high dose of *E. tenella* inoculum administered *per os* (>10,000 sporulated oocysts) led to similar oocyst load in caecal contents in germ-free and conventional chickens (Visco and Burns, 1972a). This observation may have resulted from a crowding effect resulting in maximal oocyst production. Indeed, we confirmed these findings, but we observed that with a lower inoculation dose of 1,000 sporulated oocysts administered *per os*, germ-free chickens presented a significantly lower load in oocysts in caecal contents at three different time points over a 3 days period. A lengthening of the parasite life cycle was observed and characterized by a delay in the formation of first and second generation schizonts. In germ-free chickens, at day 3.5 post-infection, second generation schizonts were small and immature with merozoites that were not formed yet while in conventional chickens, second generation schizonts were large and mature with clearly developed merozoites as revealed by histological analysis. Consequently, this delay in the asexual phase development led to a delay in the formation of gametocytes. This delay observed by histological analysis was confirmed by the expression of gametocyte specific genes, *Etgam*56 for macrogametes and *Etfoa1* for a flagellar outer arm protein 1 specific to microgametes at day 5.5 post-infection (Walker et al., 2015). This result suggests that a delay in the appearance of the gamogony stage associated to a reduced number of sexual stages most probably will result in a delayed and decreased oocyst load in germ-free chickens.

As we observed lower parasite load in caecal tissues at day 2 post-infection in germ-free chickens, we hypothesized that there could be an effect of the microbiota on the excystation step and/or in the invasion process. As excystation media used in laboratory to artificially excyst sporozoites from sporocyst contains biliary acids, we used bile from each animal. When assessing the ability of sporozoites to excyst from sporocysts, an increased excystation efficiency was observed when using bile from germ-free chickens suggesting a difference in its composition. In animals, biliary acids are synthetized from cholesterol in the liver. Conjugated bile acids (primary bile acids) are secreted in the intestine and are involved in lipid absorption. The presence of bacteria in the luminal content leads to deconjugation and dehydroxylation reactions and then to the formation of unconjugated bile acids and secondary bile acids (Long et al., 2017). Consequently, in the absence of a microbiota, the composition of the bile is different with mainly conjugated bile acids found in caeca of germ-free animals. (Madsen et al., 1976). This change in the bile composition and in particular of the biliary acids may explain the higher efficiency of excystation of sporozoites in the presence of bile from germ-free chickens. However, higher excystation efficiency in the absence of microbiota cannot explain the lower parasite load in caecal contents and in tissues found in germ-free compared to conventional chickens.

We then sought to investigate the ability of sporozoites to invade the caecal mucosa of germ-free or conventional chickens by using cloacal inoculation with sporozoites in order to shunt the excystation step. The microbiota helps in the appropriate development and maturation of the gut-associated lymphoid organs in chickens (Hegde et al., 1982). However, we observed lower parasite load in caecal contents and in tissues in germ-free chickens suggesting that the lack of maturation of the immune system in the absence of microbiota is probably not involved. Indeed, the microbiota also leads to a mature intestinal mucosa including goblet cells and their mucin production (Rolls et al., 1978) (Cheled-Shoval et al., 2014). Despite the fact that the caecal mucosa of germ-free chickens may have different characteristics, we showed that the absence of microbiota did not modify the ability of sporozoites to invade the epithelium. However, when animals were cloacally infected and kept for 7 days, the oocyst load in caecal contents and the tissue parasite load were still lower in germ-free than in conventional chickens. Even though the dose of inoculum administered orally and cloacally cannot be easily compared, cloacal infection led to a decrease in oocyst load in caecal contents similar to the one observed with oral infection. This result demonstrates that the absence of microbiota alters the development of *E. tenella* but not the capacity of sporozoites to invade caecal epithelial cells.

The microbiota harbours a wide variety of bacteria which metabolize the nutrients of the digestive content and synthetizes metabolites that can act on the immune cells (Liu et al., 2019) and/or can be critical for parasite growth. As *Eimeria* infection disrupts carbon and nitrogen metabolism (Huang et al., 2018b), the parasite is likely to require some metabolites issued at least from these two biochemical pathways. We hypothesize that some metabolites synthetized by the microbiota might be critical substrates for parasite replication and are present in reduced amount in germ-free chickens to allow its normal growth. Alternatively, these metabolites can affect parasite host cell metabolism, the lining of intestinal epithelial cells, the mucosae and indirectly the development of the parasite. Further studies will be performed to compare the precise composition of metabolites in caecal contents from germ-free and conventional chickens and to identify molecules that are important for the development of the parasite.

In conclusion, we revealed that the absence of microbiota alters the development of *E. tenella*. Strategies to modulate the composition of the microbiota and its metabolism would be of interest to inhibit parasite replication and/or to stimulate the immune response. As nutrition factors can play a major role in intestinal health and more particularly on its microbiota, it could have a beneficial effect on the outcome of the disease. Notably, innovative nutritional approaches or probiotics are societal acceptable approaches in face of growing parasite resistance to anticoccidials and lack of cost-effective prophylaxis.

## Data Availability Statement

The raw data supporting the conclusions of this article will be made available by the authors, without undue reservation.

## Ethics Statement

The animal study was reviewed and approved by CEEA Val de Loire n°19; 2018‐04‐26 (APAFIS N°13904).

## Author Contributions

FB designed the experiments. PG, GS, GF, ASa, AN, and FB performed the experiments. EG provided the conventional and germ-free chickens. CS and NL performed the bacteriological controls. AN provided the transgenic parasite Et-INV. CR, NL, TL performed the histology on tissues. FB, PG, GS analyzed the data. FB, PG, GS, SL-L, FL, CS discussed the data. FB, GS, ASi, SL-L, FL, RG, CS, wrote and/or reviewed the manuscript. All authors contributed to the article and approved the submitted version.

## Funding

This study was funded by the Région Centre Val de Loire, France (APR-IA “INTEGRITY” 2017-2019).

## Conflict of Interest

The authors declare that the research was conducted in the absence of any commercial or financial relationships that could be construed as a potential conflict of interest.

## Abbreviations

GAM56, Macrogamete specific protein; FOA1, Flagellar Outer Arm protein 1.
